# Results of Bariatric Surgery in Sana'a, Yemen, in 2019: A Prospective Cohort Study

**DOI:** 10.7759/cureus.74603

**Published:** 2024-11-27

**Authors:** Tofik A Almekhlafi, Fares S Awn, Ameera H Al-Sumat, Ebrahim M Ebrahim, Haitham M Jowah

**Affiliations:** 1 Department of Surgery, Faculty of Medicine and Health Science, Sana'a University, Sana'a, YEM; 2 Department of Surgery, University of Science and Technology Hospital, Sana'a, YEM; 3 Department of Surgery, 21 September University, Sana'a, YEM; 4 Department of Internal Medicine, 21 September University, Sana'a, YEM; 5 Family Medicine Residency Program, Department of Medical Education, Hamad Medical Corporation, Doha, QAT

**Keywords:** bariatric surgery, body mass index, diabetes mellitus, gerd, obesity, yemen

## Abstract

Background

The incidence of obesity and related comorbidities, such as diabetes, gastroesophageal reflux disease (GERD), and osteoarthritis, is increasing. Many patients with obesity do not respond to conservative treatments. For these patients, bariatric surgery, also known as metabolic bariatric surgery (MBS), has emerged as an effective option. This study aimed to evaluate the impact of laparoscopic bariatric surgery on weight loss, diabetes remission, and improvement in other comorbidities among patients in Sana'a, Yemen, in 2019.

Methods

This prospective study included 50 patients who underwent laparoscopic MBS between January and December 2019 at three tertiary hospitals in Sana’a, Yemen. The procedures included sleeve gastrectomy (SG), single anastomosis sleeve ileal bypass (SASI), and single anastomosis duodenal ileal bypass with sleeve (SADI-S). Patients were followed for six months postoperatively. Data on demographics, baseline comorbidities, and preoperative anthropometrics were collected. Weight loss was measured using multiple metrics, including percentage excess weight loss (EWL), percentage excess body mass index lost (EBMIL), and percentage total body weight loss (TBWL). Diabetes remission was defined as achieving glycated hemoglobin (HbA1c) < 6% without the use of medications.

Results

The study included 50 patients, with a mean age of 37.45 ± 10.25 years, and 28 (56%) were female. The mean preoperative weight was 121.5 ± 25.3 kg, and the mean height was 162.8 ± 8.5 cm, resulting in a mean body mass index (BMI) of 45.43 ± 7.3 kg/m². Among the patients, 15 (30%) had diabetes, 38 (76%) had GERD, and 45 (90%) had joint pain. At the six-month follow-up, the mean BMI significantly decreased to 32.46 ± 4.03 kg/m² (p < 0.001). The mean percentage of EBMIL was 63.5%, and the mean percentage of TBWL was 28.5%. Among the 15 patients with diabetes, 12 (80%) achieved diabetes remission, with HbA1c improving from 8.1 ± 2.3% to 5.3 ± 1.2% (p < 0.001). GERD improved in 30 (78.9%) of 38 patients, with complete resolution in 8 (21.1%). Joint pain improved in 39 (86.6%) of 45 patients, with complete resolution in six (13.4%).

Conclusion

Laparoscopic bariatric surgery is an effective intervention for achieving substantial weight loss, diabetes remission, and improvements in comorbidities among Yemeni patients. These findings are consistent with global literature and underscore the importance of bariatric surgery in addressing the increasing rates of obesity and metabolic disorders in Yemen. Further studies are needed to evaluate the long-term outcomes in this population.

## Introduction

The global prevalence of obesity and type 2 diabetes mellitus (T2DM) has significantly increased, presenting a dual epidemic with severe health consequences [1]. Over the years, the prevalence of obesity, particularly severe obesity, has risen dramatically, and the alarming increase in childhood obesity signals a worsening public health crisis [2]. Obesity has been consistently linked to a decreased life expectancy, contributing to millions of deaths annually worldwide. The risk of premature death escalates with an increasing body mass index (BMI), highlighting the urgent need for effective interventions [3,4].

Metabolic bariatric surgery (MBS) has emerged as a promising solution for addressing severe obesity and its associated comorbidities, including T2DM. Surgical procedures, such as Roux-en-Y gastric bypass (RYGBP), sleeve gastrectomy (SG), and duodenal switch (DS), not only result in significant weight loss but also lead to the resolution of diabetes and improvements in other obesity-related health conditions [5]. Remarkably, the beneficial effects on glycemic control are observed shortly after surgery, suggesting that mechanisms beyond weight loss contribute to these outcomes. Ghrelin, the orexigenic hormone of the gastric fundus, and incretin hormones such as glucagon-like peptide-1 (GLP-1) and peptide YY (PYY) have been identified as key players in improved glycemic control following MBS [6,7].

Understanding these mechanisms is critical for developing novel therapeutic approaches for obesity and diabetes. Factors contributing to the favorable outcomes of bariatric surgery include reduced food intake, partial malabsorption of nutrients, and anatomical alterations in the gastrointestinal tract that influence the incretin system, which plays a key role in glucose regulation. However, further research is needed to fully understand these mechanisms and to maximize their therapeutic potential.

While lifestyle interventions, such as diet therapy, behavioral modification, and exercise, are commonly employed to manage obesity, their success rates remain low, particularly in patients with severe obesity. Bariatric surgery has gained widespread attention within the medical community and among individuals with morbid obesity due to its effectiveness in achieving significant and sustained weight loss. However, concerns about the associated risks and mortality have led to reluctance in some quarters to fully embrace this treatment option.

In the Middle East and North Africa (MENA) region, bariatric surgery has become increasingly popular due to the high obesity rates in these countries. Studies from Saudi Arabia, the UAE, and Kuwait have shown positive outcomes, including substantial weight loss and improvements in metabolic health, with SG and RYGBP being the most common procedures performed [8,9]. Cultural factors, such as family influence and religious practices, may also play a role in bariatric care and outcomes in the MENA region [10]. Despite generally low complication rates and no reported mortalities in some studies, further research is needed to assess long-term outcomes and develop culture-specific care protocols for this population [11,12].

Obesity and its related comorbidities, including T2DM, gastroesophageal reflux disease (GERD), and joint pain, present significant health challenges globally. While obesity has historically been less prevalent in Yemen compared to Western countries, recent changes in lifestyle, urbanization, and dietary habits have led to a rise in obesity rates. Obesity rates in Yemen vary, with prevalence ranging from 2.5% to 12.4% in males and 12.4% to 32% in females, and central obesity affecting 24.5% of adults in Sana'a [13,14]. Among children and adolescents, overweight and obesity rates range from 8% to 23.6% [15,16]. Factors such as urban residence, higher socioeconomic status, and sedentary lifestyles are strongly associated with obesity in Yemen [17,18]. This rising obesity burden, coupled with increasing rates of T2DM, calls for effective interventions such as bariatric surgery.

Despite the growing concern over obesity in Yemen, studies assessing the efficacy of bariatric surgery in this population remain limited. This study aims to address this gap by evaluating the effectiveness of laparoscopic metabolic bariatric surgeries in patients with and without diabetes who underwent the procedure in Sana'a, Yemen, in 2019. By conducting this study, we hope to provide valuable insights into the outcomes of bariatric surgery in the Yemeni population and contribute to the broader body of knowledge on MBS.

The results of this research were posted on the preprint server Research Square on February 13, 2024.

## Materials and methods

Study design and setting

This prospective descriptive study aimed to evaluate the efficacy of bariatric surgeries on obesity and related comorbidities, including T2DM, GERD, and joint pain. It was conducted at three major tertiary hospitals in Sana'a City, Yemen, from January to December 2019. All patients provided written informed consent, and the study was conducted in accordance with the ethical standards of the hospital ethics committee and the Helsinki Declaration.

Study population

The study included patients who underwent laparoscopic MBS at three tertiary hospitals in Sana'a, Yemen, between January and December 2019. According to the 1991 National Institutes of Health (NIH) consensus guidelines [19], patients who met the following criteria were included:

BMI ≥ 40 kg/m² (severe obesity)

BMI ≥ 35 kg/m² with comorbidities such as type 2 diabetes, hypertension, or sleep apnea

Patients with a history of previous bariatric surgery, contraindications to general anesthesia, or severe psychiatric disorders were excluded from the study. Diabetes was diagnosed based on the American Diabetes Association criteria [20]. Patients were closely followed postoperatively by dieticians and surgeons to monitor their progress.

Outcome measures and definitions

The primary outcomes of the study were weight loss and diabetes remission, while the secondary outcomes included the resolution of GERD and joint pain. Diabetes remission was defined as achieving a glycated hemoglobin (HbA1c) level below 6% without the use of medications. Weight loss and diabetes remission were assessed through changes in BMI, HbA1c levels, and blood glucose levels. GERD resolution was determined by the complete absence of symptoms such as heartburn and regurgitation, while joint pain resolution was defined as the complete cessation of pain and the discontinuation of pain-relieving medications. Both GERD and joint pain outcomes were evaluated using patient-reported outcomes in combination with clinical assessments.

Surgical approach

Patients underwent preoperative assessments by an endocrinologist, anesthesiologist, and cardiologist. Once cleared, patients were scheduled for one of three types of laparoscopic metabolic bariatric surgery: SG, single anastomosis sleeve ileal (SASI) bypass, or single anastomosis duodeno-ileal bypass with sleeve (SADI-S). The type of surgery selected was based on the patients’ comorbidities and dietary behaviors.

All surgeries were performed laparoscopically using a five-port technique. In SG, a longitudinal resection of the stomach was performed starting from the antrum, 5-6 cm from the pylorus, and extending to the fundus near the cardia [21]. The remaining gastric sleeve was calibrated using a 32 French bougie. Leak testing was performed using methylene blue to ensure the integrity of the gastric sleeve.

SASI is a novel metabolic and bariatric procedure that combines sleeve gastrectomy with a side-to-side gastro-ileal anastomosis, designed to reduce gastric volume and induce malabsorption. In the SADI-S procedure, a small gastric sleeve is created by sectioning the greater curvature of the stomach, similar to SG. Subsequently, the duodenum is transected near the pylorus, and a duodenal-intestinal anastomosis is performed approximately 250 cm from the ileocecal valve, creating a common channel of 2.5 meters for nutrient absorption [22].

Statistical analysis

Data were entered and analyzed using Statistical Product and Service Solutions (SPSS, version 24.0; IBM SPSS Statistics for Windows, Armonk, NY). Descriptive statistics were used to determine baseline characteristics. Continuous data were analyzed using measures of central tendency, and categorical data were presented as percentages. Paired Student’s t-test was used to determine the mean difference of dependent variables before and after the intervention. A p-value of <0.05 was considered statistically significant.

## Results

Baseline characteristics of the patients

A total of 50 patients were included in the study, with a mean age of 37.45 ± 10.25 years. Of these, 28 (56%) were female, and 22 (44%) were male. The mean preoperative BMI was 45.43 ± 7.3 kg/m², with 76% of patients classified as having Class III obesity (BMI ≥ 40 kg/m²). Thirty percent of the patients were diagnosed with T2DM, and comorbid conditions such as GERD (76%) and joint pain (90%) were prevalent (Table 1).

**Table 1:** Baseline Characteristics of Patients SD: Standard Deviation; BMI: Body Mass Index; GERD: Gastroesophageal Reflux Disease

Characteristic	Frequency (N = 50)	Percentage (%)
Gender		
Male	22	44%
Female	28	56%
Age (years)		
Mean ± SD	37.45 ± 10.25	
BMI (kg/m²)		
Mean ± SD	45.43 ± 7.3	
Obesity Class		
Class I (30-34.9)	3	6%
Class II (35-39.9)	9	18%
Class III (≥ 40)	38	76%
Comorbidities		
Diabetes	15	30%
GERD	38	76%
Joint pain	45	90%

Effects of bariatric surgery on weight loss

The mean preoperative BMI of 45.43 ± 7.3 kg/m² significantly decreased to 32.46 ± 4.03 kg/m² at the six-month follow-up (p < 0.001). The mean percentage of EBMIL was 63.5%, indicating a significant reduction in excess BMI. Additionally, the mean percentage TBWL was 28.5%, reflecting a substantial reduction in body weight. These results highlight the effectiveness of bariatric surgery in achieving weight loss in the study population (Table 2).

**Table 2:** Effects of Bariatric Surgery on Weight Loss Note: Paired Student’s t-test was used to compare pre- and postoperative values.*Significant P value <0.05. EBMIL: % Excess BMI Lost; TBWL: % Total Body Weight Loss; BMI: Body Mass Index

Variable	Preoperative (Mean ± SD)	Postoperative (Mean ± SD)	Mean Difference	% EBMIL	% TBWL	t-value	p-value
BMI (kg/m²)	45.43 ± 7.3	32.46 ± 4.03	12.97	63.5%	28.5%	22.27	< 0.001*

Effects of weight loss and glycemic control in diabetic patients

Among the 15 patients with diabetes, there was a significant improvement in glycemic control following metabolic bariatric surgery. The majority of patients (86.7%) were treated with oral hypoglycemic drugs (OHD) preoperatively, while 6.7% were using insulin alone or in combination with OHD. The mean preoperative random blood sugar was 280.2 ± 95.2 mg/dL (range: 171-530 mg/dL), and the mean glycated hemoglobin (HbA1c) was 8.1 ± 2.3%. At the six-month follow-up, the mean HbA1c decreased significantly from 8.1 ± 2.3% preoperatively to 5.3 ± 1.2% postoperatively, with a mean difference of 2.8% (p < 0.001). Additionally, random blood glucose levels improved significantly, with a mean reduction of 164.8 mg/dL (p < 0.001).

Among the two patients who were using insulin preoperatively, one patient (50%) stopped insulin therapy entirely, while the other, who had been using 90 units of insulin per day, reduced the dosage to 40 units per day. At six months postoperatively, 80% of the patients with diabetes achieved diabetes remission (HbA1c < 6% without medication). This improvement was confirmed through patient interviews and clinical assessments at follow-up (Table 3). All patients who were using OHD preoperatively stopped their medications following surgery after achieving glycemic control.

**Table 3:** Effects of Glycemic Control in Diabetic Patients (N = 15) Note: *Significant P value <0.05. Paired Student’s t-test was used to compare pre- and postoperative values. HbA1c: Glycated Hemoglobin; RBS: Random Blood Sugar

Variable	Preoperative (Mean ± SD)	Postoperative (Mean ± SD)	Mean Difference	t-value	p-value
HbA1c (%)	8.1 ± 2.3	5.3 ± 1.2	2.8	4.9	< 0.001*
RBS (mg/dL)	280.2 ± 95.2	111.4 ± 39	164.8	8.0	< 0.001*

GERD and joint pain improvement

Among the 38 patients who had GERD preoperatively, 30 (78.9%) reported improvement in symptoms, while eight (21.1%) experienced complete resolution of their GERD symptoms. For the 45 patients with preoperative joint pain, 39 (86.6%) reported overall improvement, and six (13.4%) experienced complete resolution of joint pain.

When examining the outcomes based on the type of surgical procedure, patients who underwent SG had a slightly lower rate of GERD symptom improvement compared to those who underwent SASI or SADI-S. This difference may be attributed to SG being a relative contraindication for GERD due to its potential to increase intra-gastric pressure, which can exacerbate GERD symptoms. In contrast, patients who underwent SASI and SADI-S procedures had a higher rate of GERD symptom improvement, likely due to their malabsorptive components and anatomical alterations in the gastrointestinal tract, which can reduce GERD symptoms.

Regarding joint pain, 39 (86.6%) of the 45 patients reported improvement, with six (13.4%) achieving complete resolution. The rates of joint pain improvement were similar across all surgical techniques, indicating that bariatric surgery, regardless of the procedure type, was effective in alleviating joint pain related to obesity. However, there were no significant differences between SG, SASI, and SADI-S in terms of joint pain resolution, suggesting that weight loss itself is a major factor in improving musculoskeletal pain.

The outcomes of GERD and joint pain improvement based on surgical techniques are summarized in Table 4.

**Table 4:** Improvement in GERD and Joint Pain After Surgery Note: *Significant P value <0.05. A binomial test was used to assess the improvement and resolution of symptoms. GERD: Gastroesophageal Reflux Disease

Condition	Preoperative N (%)	Improved (%)	Resolved (%)	p-value
GERD (N = 38)	38 (100%)	30 (78.9%)	8 (21.1%)	< 0.001
Joint Pain (N = 45)	45 (100%)	39 (86.6%)	6 (13.4%)	0.001

## Discussion

Our study demonstrated a significant reduction in BMI following laparoscopic bariatric surgery, with preoperative BMI decreasing from 45.43 ± 7.3 kg/m² to 32.46 ± 4.03 kg/m² postoperatively (p < 0.001). The % EBMIL was 63.5%, and the % TBWL was 28.5%. These findings align with global trends observed in bariatric surgery outcomes. For instance, a systematic review by O’Brien et al. reported similar outcomes, showing that patients typically achieve an average EBMIL of 60%-70% and a TBWL of 25%-30% following surgery, with variations depending on procedure type and follow-up period [23].

Similarly, Peterli et al. found that patients undergoing LSG or RYGB experienced comparable TBWL of around 25% after one year [24]. Our findings, with a TBWL of 28.5%, are in line with these studies, indicating that, despite geographic and demographic differences, the results of bariatric surgery on weight reduction are globally consistent.

One of the most significant findings in our study is the high diabetes remission rate of 80% in the diabetic cohort. The mean HbA1c decreased significantly from 8.1 ± 2.3% preoperatively to 5.3 ± 1.2% postoperatively (p < 0.001), with a reduction in RBS by 164.8 mg/dL (p < 0.001). This is supported by other studies demonstrating the effectiveness of bariatric surgery in achieving diabetes remission. For example, Schauer et al. found that up to 85% of patients achieved diabetes remission following RYGB or LSG, particularly those with a shorter duration of diabetes prior to surgery [25].

The American Diabetes Association (ADA) recommends bariatric surgery for managing T2DM in severely obese patients, especially those with a BMI ≥ 35 kg/m² who struggle to control hyperglycemia through medical therapy [20]. This aligns with our findings, which show significant improvements in glycemic control post-surgery in a population that met these criteria.

Our study found that 78.9% of patients experienced improvement in postoperative GERD symptoms, while 21.1% reported complete resolution. However, we did not specifically stratify GERD outcomes for patients who underwent SG in this study. Although GERD improvement is commonly reported following RYGB, SG has been associated with the onset or worsening of GERD symptoms in some patients. As noted by Nguyen et al., while SG is effective in reducing weight, it can lead to de novo GERD in certain individuals due to anatomical changes that increase intra-gastric pressure [26]. Therefore, preoperative GERD is generally considered a contraindication for SG, as this procedure can exacerbate GERD symptoms. Kehagias et al. also observed that SG can worsen GERD and even lead to new-onset reflux in some patients, making careful patient selection critical when SG is considered for individuals with pre-existing GERD [21].

Given this, the improvement in GERD symptoms observed in our cohort may mask any distinct differences for SG patients. Future studies should focus on stratifying GERD outcomes by surgical procedure to better understand the specific impact of SG on GERD symptoms.

Conversely, Xu et al. suggested that RYGB may be a more appropriate option for patients with pre-existing GERD, as it has a higher likelihood of resolving GERD symptoms [27]. In our study, the significant improvement in GERD symptoms across the cohort suggests that SG may still benefit some patients, but careful patient selection is essential to minimize the risk of exacerbating GERD.

Our study showed that 86.6% of patients reported improvement in joint pain, with 13.4% reporting complete resolution. The positive impact of bariatric surgery on musculoskeletal health is well documented. For example, Groen et al. found a 37% reduction in knee pain among obese patients who underwent bariatric surgery [28]. Hacken et al. similarly reported improvements in joint pain and physical function, particularly in patients with obesity-related osteoarthritis [29].

The weight reduction following bariatric surgery reduces mechanical strain on weight-bearing joints, leading to pain relief and enhanced mobility. This highlights the dual benefits of bariatric surgery in both weight reduction and the improvement of comorbid conditions such as joint pain.

One notable contribution of this study is its context within Yemen, a country previously considered to have a lower prevalence of obesity and metabolic syndrome [30]. However, as obesity rates continue to climb worldwide, Yemen is no exception. This study brings attention to the growing obesity and diabetes problem in the country, emphasizing the need for increased awareness and accessible treatment options. While bariatric surgery is a well-documented intervention globally, its application in Yemen highlights the potential for surgical solutions to be integrated into the broader public health strategies for combating the obesity pandemic in this region.

The strengths of this study include its prospective design, rigorous follow-up, and comprehensive assessment of both primary and secondary outcomes. However, we acknowledge several limitations. One notable limitation is the analysis of outcomes from multiple types of bariatric procedures performed at three different hospitals, which may introduce variability in the data due to differences in surgical techniques, patient management, and hospital practices. Additionally, the sample size was relatively small, which may limit the generalizability of the findings to a wider population. The follow-up period was also relatively short, which limits our ability to assess the long-term sustainability of weight loss and diabetes remission. Further studies with larger sample sizes, longer follow-up periods, and more robust comparative analyses of different bariatric procedures are needed to provide more comprehensive data and evaluate the long-term effectiveness of bariatric surgery in diverse settings.

## Conclusions

Our findings align with existing literature and demonstrate the significant efficacy of bariatric surgery in achieving weight loss, diabetes remission, and the resolution of comorbidities, such as GERD and joint pain. These results highlight the effectiveness of SG, SASI, and SADI-S in addressing obesity and its complications, reinforcing bariatric surgery as a viable treatment option in populations with rising obesity rates, including Yemen. Further research with larger cohorts and longer follow-up periods is necessary to explore the long-term benefits and potential challenges of bariatric surgery in this region.
